# Autonomic Dysfunction in Myalgic Encephalomyelitis/Chronic Fatigue Syndrome (ME/CFS): Findings from the Multi-Site Clinical Assessment of ME/CFS (MCAM) Study in the USA

**DOI:** 10.3390/jcm14176269

**Published:** 2025-09-05

**Authors:** Anindita Issa, Jin-Mann S. Lin, Yang Chen, Jacob Attell, Dana Brimmer, Jeanne Bertolli, Benjamin H. Natelson, Charles W. Lapp, Richard N. Podell, Andreas M. Kogelnik, Nancy G. Klimas, Daniel L. Peterson, Lucinda Bateman, Elizabeth R. Unger

**Affiliations:** 1 Division of High-Consequence Pathogens and Pathology, Centers for Disease Control and Prevention, Atlanta, GA 30329, USA; lxo4@cdc.gov (A.I.); yolandachen654@gmail.com (Y.C.); attell_jacob@bah.com (J.A.); dejones@umich.edu (D.B.); jeannebertolli@gmail.com (J.B.); eru0@cdc.gov (E.R.U.); 2 Booz Allen Hamilton, Inc., Atlanta, GA 30309, USA; 3 Department of Neurology, Icahn School of Medicine at Mount Sinai, New York, NY 10029, USA; benjamin.natelson@mountsinai.org; 4 Hunter-Hopkins Center, Charlotte, NC 28226, USA; cwlapp@drlapp.net; 5 Richard N. Podell Medical, Summit, NJ 07901, USA; 6 ProDx Health, Menlo Park, CA 94025, USA; andy@y-dna.com; 7 Institute for Neuro Immune Medicine, Miami, FL 33183, USA; nklimas@nova.edu; 8 VA Medical Center, Geriatric Research and Education Clinical Center, Miami, FL 33125, USA; 9 Sierra Internal Medicine, Incline Village, NV 89451, USA; dpeterson@sierrainternalmed.com; 10 Bateman Horne Center, Salt Lake City, UT 84102, USA; lbateman@batemanhornecenter.org

**Keywords:** dysautonomia, myalgic encephalomyelitis/chronic fatigue syndrome (ME/CFS), infection-associated chronic conditions and illnesses (IACCIs), orthostatic intolerance (OI), postural orthostatic tachycardia syndrome (POTS), orthostatic hypotension (OH), Composite Autonomic Symptoms Scale 31 (COMPASS-31), Patient Reported Outcome Measurement Information System (PROMIS), National Aeronautics and Space Administration (NASA) Lean Test

## Abstract

**Background/Objectives**: Symptoms of autonomic dysfunction are common in infection-associated chronic conditions and illnesses (IACCIs), including myalgic encephalomyelitis/chronic fatigue syndrome (ME/CFS). This study aimed to evaluate autonomic symptoms and their impact on ME/CFS illness severity. **Methods**: Data came from a multi-site study conducted in seven ME/CFS specialty clinics during 2012–2020. Autonomic dysfunction was assessed using the Composite Autonomic Symptom Scale 31 (COMPASS-31), medical history, and a lean test originally described by the National Aeronautics and Space Administration (NASA). Illness severity was assessed using Patient-Reported Outcomes Measurement Information System measures, the 36-item short-form, as well as the CDC Symptom Inventory. This analysis included 442 participants who completed the baseline COMPASS-31 assessment, comprising 301 individuals with ME/CFS and 141 healthy controls (HC). **Results**: ME/CFS participants reported higher autonomic symptom burden than HC across three assessment tools (all *p* < 0.0001), including the COMPASS-31 total score (34.1 vs. 6.8) and medical history indicators [dizziness or vertigo (42.6% vs. 2.8%), cold extremities (38.6% vs. 5.7%), and orthostatic intolerance (OI, 33.9% vs. 0.7%)]. Among ME/CFS participants, 97% had at least one autonomic symptom. Those with symptoms in the OI, gastrointestinal, and pupillomotor domains had significantly higher illness severity than those without these symptoms. **Conclusions**: ME/CFS patients exhibit a substantial autonomic symptom burden that correlates with greater illness severity. Individualized care strategies targeting dysautonomia assessment and intervention may offer meaningful improvements in symptom management and quality of life for those with ME/CFS and similar chronic conditions.

## 1. Introduction

Symptoms of autonomic dysfunction are common in infection-associated chronic conditions and illnesses (IACCI), including myalgic encephalomyelitis/chronic fatigue syndrome (ME/CFS) [1]. Autonomic dysfunction is among the proposed pathologies that may contribute to core symptoms of ME/CFS, which is a debilitating, long-term illness that affects multiple body systems. However, dysautonomia is not unique to ME/CFS. Dysautonomia is an overarching term that describes multiple symptoms and disorders involving the autonomic nervous system, including orthostatic intolerance (OI), heart rate dysregulation, gastrointestinal dysmotility, fatigue, bladder dysfunction, abnormal sweating, and others [2]. OI is included in the 2015 Institute of Medicine diagnostic criteria for ME/CFS [3] and is one of the most common presenting clinical conditions seen in those with autonomic dysfunction. The heterogeneity of ME/CFS limits the ability to identify a consistent pattern of autonomic dysfunction across affected individuals. To address this variability, researchers have proposed ME/CFS subtypes based on the presence or absence of specific autonomic abnormalities, such as postural orthostatic tachycardia syndrome (POTS). Autonomic function is typically evaluated through a range of methods—including heart rate variability analysis, tilt table testing, and self-reported questionnaires—although these tools vary in their application and results are not always strongly correlated across studies [3].

The Composite Autonomic Symptom Scale 31 (COMPASS-31), originally derived from the Autonomic Symptom Profile, is a relatively short but broad self-assessment instrument for autonomic symptoms and function in six domains covering several organ systems (orthostatic intolerance, pupillomotor, vasomotor, secretomotor, gastrointestinal, and bladder) that can be used as a clinical screening tool for dysautonomia [4]. The COMPASS-31 has been used, with varying degrees of validation, to screen for autonomic symptoms experienced by people with a variety of conditions, such as multiple sclerosis, Parkinsonism, compressive myelopathy, diabetes, and small fiber polyneuropathy [5,6,7,8,9,10,11].

Studies of OI in people with ME/CFS, including postural orthostatic intolerance syndrome (POTS), neurally mediated hypotension (NMH), or orthostatic hypotension (OH), using varied methods report a wide range (30–70%) in the prevalence of dysautonomia [3]. Research on dysautonomia overall in ME/CFS has most recently focused on activity-related data like heart rate variability and patient-reported outcome measures, such as the COMPASS-31 [12,13,14,15,16]. These studies have indicated that dysautonomia of varying kinds can be identified in people with ME/CFS but have not directly examined the association of dysautonomia with symptom burden and functioning. The present study aims to evaluate dysautonomia among a cohort of patients with ME/CFS seen in seven different US specialty clinics using clinical history, the COMPASS-31, and the Lean Test [17], as well as to evaluate whether the presence of any dysautonomia affects illness severity among people with ME/CFS.

## 2. Materials and Methods

### 2.1. Data Source and Study Sample

Data came from the Multi-Site Clinical Assessment of Myalgic Encephalomyelitis/Chronic Fatigue Syndrome (MCAM) study [18,19]. The study was approved by the Institutional Review Boards of the Centers for Disease Control (CDC) and Prevention, Open Medicine Institute (OMI) Consortium, Mount Sinai Beth Israel (MSBI), and Nova Southeastern University. One of the objectives for the MCAM study was to use standardized instruments to measure ME/CFS illness domains, including autonomic symptoms. In brief, MCAM was conducted in multiple stages with a rolling cohort design between 2012 and 2020. Participants were enrolled at different stages; therefore, the baseline data correspond to the stage at which each participant was first enrolled. Recruitment was carried out across seven ME/CFS specialty clinics in the United States. Included ME/CFS patients were aged 18–70 years at their baseline enrollment and had been diagnosed with CFS, ME, or post-infectious fatigue, or managed as other ME/CFS patients in the clinical practice. Each clinician determined patient eligibility using their clinical expertise with the illness; patients were not required to fit a specific case definition. The exclusion criteria included onset of illness at an age older than 62 years, human immunodeficiency virus infection, current pregnancy, and dementia. Healthy controls (HCs) were recruited from neighborhoods near participating ME/CFS specialty clinics through health screening events and flyer distribution. Individuals who expressed interest were eligible to participate as HCs if, in a brief health screening questionnaire, they reported fewer than 10 physically or mentally unhealthy days in the past 30 days.

This paper focuses on 442 participants (301 ME/CFS and 141 HC) who completed their first COMPASS-31 assessment, a key measure of autonomic symptoms.

### 2.2. Measures

#### 2.2.1. Autonomic Dysfunction Assessment

In this paper, autonomic dysfunction was evaluated using three assessment tools: (1) the Composite Autonomic Symptom Scale 31 (COMPASS-31) for six autonomic dysfunction domains; (2) a medical history review, mainly for OI and autonomic symptoms; and (3) the Lean Test for two forms of OI (POTS and OH).

The Composite Autonomic Symptom Scale 31 (COMPASS-31): The COMPASS-31 consists of 31 questions that explore symptoms related to each of the six autonomic domains, including the OI (4 items), pupillomotor (5 items), vasomotor (3 items), secretomotor (4 items), gastrointestinal (GI) (12 items), and bladder (3 items) domains [4,10]. The possible score ranges for each domain are as follows: OI (0–40), pupillomotor (0–5), vasomotor (0–5), secretomotor (0–15), GI (0–25), and bladder (0–10), with a cumulative total score of 0–100. Higher scores reflect more severe autonomic symptoms. Different cut-off scores, indicating elevated risk of autonomic dysfunction, have been applied across the six domains of the COMPASS-31 questionnaire. Singh et al. (2019) reported strong sensitivity and specificity for cut-off scores of 14 and 5.8 in the orthostatic intolerance and gastrointestinal domains, respectively, based on a tertiary sample of 54 patients with type 2 diabetes mellitus [11]. For the remaining four domains, cut-off values were derived from Adler et al. (2018), who studied individuals with diabetic polyneuropathy: pupillomotor (2.2), vasomotor (1.0), secretomotor (5.5), and bladder (1.6) [20]. Their research also included comparisons with other populations, such as individuals diagnosed with scleroderma, fibromyalgia, and small-fiber polyneuropathy. Although our study did not focus on determining a cut-off for the COMPASS-31 total score, it is noteworthy that various cut-off values have been employed depending on the specific clinical context and target population.

Medical History: To minimize the burden of the study on participants, authorized study personnel reviewed participants’ medical records to abstract information using a standardized medical history form [18]. The form included fields for information on the review of organ systems. Medical record information of interest in this paper included dysautonomia, or autonomic-related symptoms of dizziness or vertigo, irregular heartbeats or palpitations, sweating, cold extremities (“feet or hands get cold very easily”), constipation, dry mouth, low blood pressure, shortness of breath, poor appetite, malabsorption or chronic diarrhea, trouble emptying the bladder, or fainting.

Lean Test: Using a lean rather than an upright posture was first proposed in 1985 and then used by one of our clinical sites [17,21] as part of a physical examination protocol originally developed by the National Aeronautics and Space Administration (NASA) and later standardized for the MCAM study. Specifically, after the patient had been lying supine for at least 10 min, baseline physiological measures—systolic/diastolic blood pressure (SBP/DBP) and heart rate—were recorded twice, one minute apart. Participants were then asked to stand with legs together approximately 6–8 inches from a wall and then to lean against the wall for 10 min while heart rate and blood pressure measurements were taken every minute. Orthostatic hypotension (OH) was defined as a decrease of SBP ≥20 mmHg from supine levels occurring on at least 2 determinations after leaning. POTS was defined as an increase of HR ≥30 beats per minute (BPM) during leaning compared with resting or ≥120 BPM in the absence of OH.

#### 2.2.2. Illness Severity Assessment

Illness severity was assessed within four focus areas: (1) symptom-oriented measures (CDC Symptom Inventory (CDC-SI)) [22,23]; (2) domain-specific measures: fatigue (PROMIS Fatigue), pain (PROMIS Pain Behavior and Pain Interference), and sleep (PROMIS Sleep Disturbance and Sleep-Related Impairment) [23,24,25,26,27,28,29]; (3) an OI function measure: Orthostatic Grading Scale (OGS), a self-assessment tool used to measure the severity, frequency, and interference with daily activities for symptoms of OI due to hypotension [30]; and (4) function-oriented measures: well-being and functioning (36-item Short Form Health Survey (SF-36v2)) [31], containing subscales for physical functioning, role physical, bodily pain, vitality, general health, role emotional, social functioning, and mental health. The index measure in this paper, COMPASS-31, was added to the MCAM study at a later stage; therefore, we examined the impact of loss to follow-up by showing overall functioning and symptom status of ME/CFS participants at intake in Table S1. In general, the follow-up ME/CFS cohort used in this analysis was not significantly different from the initial cohort reported in Unger et al. 2017 [18].

Other measures include sample characteristics such as age, sex, race/ethnicity, marital status, employment, health insurance coverage, education level, number of office visits in the past year, body mass index (BMI), illness duration, and illness onset status. Additionally, we assessed the impact of other comorbidities on the relationship between dysautonomia and ME/CFS by analyzing data on ongoing conditions abstracted from medical records, covering a total of 150 conditions and illnesses. Details on the medical history form used for this evaluation are available in the Supplemental Materials of Unger et al. (2017): https://pmc.ncbi.nlm.nih.gov/articles/instance/5565838/bin/NIHMS891755-supplement-supplemental_files.pdf (accessed on 15 August 2025) [18]. To quantify comorbidity burden, we created an aggregated variable representing the total number of conditions or illnesses that patients were still experiencing.

### 2.3. Statistical Analysis

Descriptive statistics were shown as means and standard deviations (SD) for continuous variables and frequencies and percentages for categorical variables. Comparisons between groups were evaluated using χ^2^ tests or Fisher’s exact tests for categorical variables. The normality of continuous variable distributions was evaluated using the combination of the Shapiro–Wilk test and Q-Q plots, and depending on the distribution normality of the analyzed variables, the *t*-test (normal) or the Mann–Whitney U test (non-normal) was used to evaluate differences in measured values between groups. We used logistic regression for binary outcomes (e.g., presence/absence of autonomic dysfunction), Poisson regression for count data (e.g., number of types of autonomic dysfunction), and linear regression for continuous outcomes. Effect sizes were calculated as the magnitude of the differences (Cohen’s d for mean differences (∆) or Cohen’s h for percentage differences). Cohen’s effect size is interpreted as a “small” effect (0.2), a “moderate” effect (0.5), a “large” effect (0.8), or a “very large” effect (1.2) [32]. Analyses were performed using SAS software, version 9.4 (SAS Institute Inc., Cary, NC, USA), and the level of significance was set at *p* < 0.05.

## 3. Results

Socio-demographic and clinical characteristics are summarized in Table 1. The majority of participants in both groups were female (ME/CFS: 69.8% vs. HC: 70.9%, *p* = 0.8048). Participants with ME/CFS were older than the HCs (51.4 vs. 47.0 years, *p* = 0.0012) and more likely to identify as White and non-Hispanic (ME/CFS vs. HC: White: 90.4% vs. 56.0%, *p* < 0.0001; non-Hispanic: 84.1% vs. 61.7%, *p* < 0.0001). ME/CFS participants were also more likely to have health insurance (96.3% vs. 87.2%, *p* = 0.0001) and reported a higher number of clinic office visits (5.1 vs. 0.7 visits, *p* < 0.0001). Conversely, ME/CFS participants were less likely to be employed (19.9% vs. 75.8%, *p* < 0.0001) or to be obese (11.3% vs. 26.2%, *p* < 0.0001). No statistically significant differences between ME/CFS and HC individuals were observed in terms of marital status or educational attainment. Among ME/CFS participants, the mean duration of illness was 18.4 ± 10.98 years, with 63.1% reporting a sudden onset of illness.

Table 2 highlights the autonomic symptoms by group. COMPASS-31 total scores, as well as domain-specific scores, were significantly higher in ME/CFS participants than in HCs (*p* < 0.0001). Effect sizes for group mean differences in six domain scores ranged from very large in the pupillomotor domain (d = 1.75) to moderate in bladder and vasomotor domains (d = 0.67). Additionally, ME/CFS participants demonstrated significantly higher OGS scores than HCs (8.3 vs. 0.6, *p* < 0.0001, Cohen’s d = 1.86).

According to medical records, autonomic-related symptoms or abnormalities were significantly higher in ME/CFS participants than HCs (*p* < 0.01), with the exception of “Other Dysautonomia.” Large Cohen’s h effect sizes were observed for the proportion of participants having autonomic-related symptoms, including dizziness or vertigo (42.6% vs. 2.8%, h = 1.08), cold extremities (38.6% vs. 5.7%, h = 0.86), OI (33.9% vs. 0.7%, h = 1.07), irregular heartbeat or palpitations (29.9% vs. 2.8%, h = 0.82), and dry mouth (27.5% vs. 2.1%, h = 0.81). Moderate effect sizes were noted in other symptoms, such as constipation (28.2% vs. 2.8%, h = 0.78) and low blood pressure (22.5% vs. 3.5%, h = 0.61).

**Figure 1:**
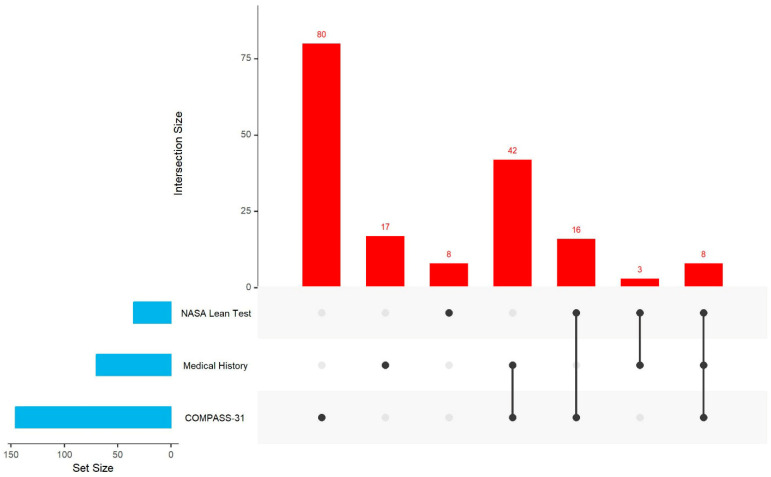
UpSet plot for OI in people with myalgic encephalomyelitis/chronic fatigue syndrome (ME/CFS) who completed all three assessment tools (*n* = 219). Note: UpSet shows intersections in a matrix, with the rows corresponding to the three assessment tools (medical history, NASA Lean Test, and COMPASS-31) and the columns corresponding to the intersections between these tool sets. The size of the sets and the overlap of these three sets are shown as bar charts in blue and red.

Among three assessment tools (COMPASS-31, medical history, and Lean Test), nearly all ME/CFS participants (97%) were identified as having one or more autonomic abnormalities. In contrast, only 38% of the healthy controls had autonomic abnormalities. Among 301 ME/CFS participants, 282 (93.7%) were identified by COMPASS-31 as having any of the six autonomic dysfunction risks: OI (66.8%), GI (66.2%), pupillomotor (57.3%), secretomotor (52.7%), bladder (29.8%), and vasomotor (27.8%) (Table 3). Notably, 181 (64.2%) participants identified by COMPASS-31 as having risk of dysfunction had no documented ongoing autonomic abnormalities in their medical history reviews. However, 22 of them were later confirmed through the Lean Test to have POTS or OH. Since OI was the only autonomic dysfunction assessed by all three tools, we examined their concordance in identification. Of 219 ME/CFS participants who had data from all three assessment tools, 174 had OI identified by at least one method; 60.3% (105) had OI identified by one tool only (80 by COMPASS-31, 17 by medical history, and 8 by the NASA Lean Test), and 39.7% (69) had OI identified by two or three tools (Figure 1).

Autonomic symptoms significantly contributed to both symptom burden and functional impairment in ME/CFS participants, as evidenced by associating these measures with the COMPASS-31 dysautonomia risk. These scores revealed notable differences between ME/CFS participants with and without a risk of dysautonomia (Table 3 and Table 4).

As expected, measures addressing similar concepts showed large effects (d >= 0.8): OGS scores demonstrated a substantial difference between those with OI risk and those without (d = 1.11, Table 3), while CDC-SI “sensitivity to light” scores differed significantly between those with pupillomotor risk and those without (d = 1.26, Table 4).

OI risk showed a significant effect on fatigue-related measures—[PROMIS Fatigue (∆ = 6.0, d = 0.81), SF-36 Vitality (∆ = 6.0, d = 0.66), CDC-SI “fatigue after exertion” (∆ = 3.5, d = 0.63)], sleep measures, [PROMIS Sleep Impairment (∆ = 5.4, d = 0.73)], and measures of function [SF-36 Physical Functioning (∆ = 6.6, d = 0.64) and Social Functioning (∆ = 6.2, d = 0.53)]. Pupillomotor risk showed a significant effect on pain-related measures [SF-36 Bodily Pain (∆ = 7.4, d = 0.73), PROMIS Pain Interference (∆ = 6.4, d = 0.71) and Pain Behavior (∆ = 4.3, d = 0.59), CDC-SI “muscle aches and pains” (∆ = 3.6, d = 0.70), and “joint pain” (∆ = 2.8, d = 0.56)], as well as measures of function [SF-36 general health (∆ = 5.6, d = 0.65) and SF-36 Physical Functioning (∆ = 6.3, d = 0.60)], cognitive measures [CDC-SI “concentration” (∆ = 3.3, d = 0.64), “memory problems” (∆ = 2.9, d = 0.59)], fatigue [PROMIS Fatigue (∆ = 4.7, d = 0.63)], and OGS (∆ = 2.5, d = 0.53). GI risk showed a significant effect on sleep-related measures [PROMIS Sleep Impairment (∆ = 4.4, d = 0.58), CDC-SI “sleep problems” (∆ = 2.7, d = 0.51) and “unrefreshing sleep” (∆ = 2.6, d = 0.50)]. After adjusting for confounding factors (age, sex, illness duration, and the number of ongoing comorbidities), the dysautonomia risks identified by COMPASS-31 still showed a statistically significant impact on ME/CFS-related symptoms and functioning.

## 4. Discussion

We found that people with ME/CFS exhibited a higher burden of autonomic symptoms than healthy controls, as determined through both clinical evaluation (NASA Lean Test) and patient-reported measures (medical records and COMPASS-31). These findings align with previous studies documenting dysautonomia or autonomic dysfunction in people with ME/CFS [21,33,34,35]. However, most prior research has relied on a single assessment tool to evaluate autonomic dysfunction or its subtype, orthostatic intolerance (OI).

In contrast, our study used multiple assessment tools to reveal that nearly all ME/CFS participants (97%) had one or more autonomic abnormalities. Reviewing a patient’s medical history is a routine clinical practice that can serve as a valuable tool for monitoring both past and ongoing medical issues. However, we lack information regarding whether the dysautonomia recorded in medical records was confirmed through objective clinical tests, such as tilt table tests, cardiovascular autonomic reflex tests, or heart rate variability assessments. It is important to note that some objective tests can be costly and may require out-of-pocket payments, adding to the financial burden of ME/CFS patients, who already bear a substantial portion of their medical expenses personally [36]. To address this challenge, accessible screening tools like COMPASS-31, a shortened version of the 73-item COMPASS, could be highly beneficial. COMPASS has been proven to be a valid screening tool for autonomic dysfunction [33]. More affordable objective tests, such as the Lean Test, could also help reduce financial barriers. Our study demonstrated that the Lean Test is very useful in identifying additional ME/CFS patients with POTS or OH who were not previously documented in medical records for these conditions. Furthermore, additional presentations of OI can also be identified using the NASA Lean Test with capnography, as implemented at one of the MCAM study sites [21].

By leveraging available tools like the Lean Test, COMPASS-31, and patient medical history, clinicians can enhance the diagnosis of OI and other autonomic dysfunctions. The clinical heterogeneity of patients with ME/CFS and/or dysautonomia often leads to delays in diagnosis. Employing these tools could help reduce those delays, ensuring that patients receive timely support for symptom management.

People with ME/CFS who also had dysautonomia risks identified in COMPASS-31 exhibited an increased symptom burden and poorer functioning compared to those without risks, particularly in relation to OI, GI issues, and pupillomotor dysfunction. Specifically, the co-occurrence of the OI, pupillomotor, and GI risks had a substantial impact on orthostatic grading scale (OGS) scores, sensitivity to light, and diarrhea, respectively, as they measured similar concepts. Although research into the influence of autonomic dysfunction on ME/CFS severity remains limited, existing studies have reported its impact on worsening function, particularly when OI, secretomotor, and GI dysfunction co-occur with other conditions [19,37,38]. Our study further supports the impact of autonomic dysfunction on ME/CFS illness severity across multiple domains. We found that both OI and pupillomotor risks are significantly associated with increased fatigue and greater impairments in physical functioning among people with ME/CFS. Additionally, sleep-related impairment in people with ME/CFS appears to be further aggravated by both OI and GI risks, a relationship supported by prior research [39,40]. Circadian rhythm misalignment, recognized as a key factor in sleep disturbances [41,42], may contribute to these disruptions through mechanisms involving wakefulness-related neuropeptide orexin [42]. Furthermore, our findings indicate a significant impact of the pupillomotor risk on pain and cognitive impairment, while OI further intensified the post-extensional malaise (PEM). The association of autonomic dysfunction with pain, cognitive impairment, and PEM has not been extensively investigated in people with ME/CFS. These findings reinforce the notion that autonomic dysregulation may further contribute to the level of severity in fatigue, sleep disturbances, cognitive function, pain, PEM, and functional impairment in those with ME/CFS.

Utilizing available pharmacologic and non-pharmacologic treatment options for dysautonomia holds promise in alleviating the autonomic symptom burden in people with ME/CFS [43,44,45,46,47]. Additionally, our findings highlight the need for further research into clinically significant aspects of dysautonomia, particularly subgroup analyses, to refine treatment and management approaches [48].

### Limitations

A key strength of this study is its use of data from seven ME/CFS specialty clinics across the US, resulting in a larger sample size than previous studies on similar topics. However, the study sample may not be fully representative of the broader ME/CFS population, as most participants were White, had higher levels of education, and had access to health insurance. Other limitations include the subjectivity of several patient-reported measures used to assess dysautonomia. While validated questionnaires, such as the COMPASS-31 and OGS, are useful for evaluating autonomic dysfunction, relying on the COMPASS-31 instead of the 169-item Autonomic Symptom Profile is inherently a limitation, as the COMPASS-31 does not assess syncope, sexual dysfunction, or sleep dysfunction. Furthermore, some symptoms or conditions noted in medical history reviews are not necessarily specific to dysautonomia and may be attributed to other illnesses. For example, shortness of breath may be due to lung pathology rather than low cardiac output associated with autonomic dysfunction. Medical examinations and documentation in the medical record were not standardized, so the absence of information in the record cannot be assumed to mean the absence of a symptom or condition in the patient. Nevertheless, our assessment of medical history demonstrates that even routine clinical questions, along with survey instruments, can help healthcare providers identify potential autonomic involvement in patients with ME/CFS. While syncope (including vasovagal syncope) is not assessed using the COMPASS-31, individualized evaluation can include addressing this, and treatment related to parasympathetic (vagal) modulation could be considered.

Recent innovations in autonomic testing technologies are driving the development of more sensitive, less invasive approaches to evaluating the autonomic nervous system. Key advancements include enhanced techniques for assessing sudomotor function, more precise methods for analyzing heart rate variability, and the integration of neuroimaging to visualize autonomic nerve pathways. There is also a growing emphasis on improving accessibility and patient comfort, with new testing protocols designed to support telemedicine and remote monitoring applications [49,50,51,52]. However, the absence of these data in our source study limits the ability to examine this topic in depth. Future research should aim to address this gap.

## 5. Conclusions

Our study revealed consistent and robust differences in dysautonomia burden between patients with ME/CFS and healthy controls, as assessed using clinical history and multiple assessment tools. Dysautonomia abnormalities, as captured by the COMPASS-31 domains, were associated with greater illness burden and functional impairment. Our results highlight the potential value of recognizing and addressing dysautonomia symptoms in ME/CFS to reduce the illness severity. Future studies are necessary to confirm whether individualized treatment strategies can effectively improve the quality of life for this population.

## Figures and Tables

**Table 1:** Sample characteristics by study group (*n* = 442): myalgic encephalomyelitis/chronic fatigue syndrome (ME/CFS) and healthy control (HC).

	ME/CFS (*n* = 301)	HC (*n* = 141)	*p*-Value
Age (yrs), mean (SD)	51.4 (13.30)	47.0 (13.83)	0.0012
Female, *n* (%)	210 (69.8)	100 (70.9)	0.8048
Race, *n* (%)			<0.0001
White	272 (90.4)	79 (56.0)	
Black/African American	1 (0.3)	16 (11.3)	
All others	14 (4.7)	33 (23.4)	
Missing	14 (4.7)	13 (9.2)	
Ethnicity, *n* (%)			<0.0001
Hispanic	14 (4.7)	44 (31.2)	
Non-Hispanic	253 (84.1)	87 (61.7)	
Missing	34 (11.3)	10 (7.1)	
Marital Status, *n* (%)			0.5363
Married/committed	160 (53.2)	78 (55.3)	
Previously married	52 (17.3)	29 (20.6)	
Never married	86 (28.6)	32 (22.7)	
Missing	3 (1.0)	2 (1.4)	
Employment, *n* (%)			<0.0001
Full-time	38 (12.6)	81 (57.4)	
Part-time	22 (7.3)	26 (18.4)	
Not working	205 (68.1)	23 (16.3)	
Missing	36 (12.0)	11 (7.8)	
Had insurance, *n* (%)	290 (96.3)	123 (87.2)	0.0001
Education, *n* (%)			0.2449
Less than high school	1 (0.3)	2 (1.4)	
High school graduate	66 (21.9)	39 (27.7)	
College graduate	123 (40.9)	50 (35.5)	
Post-college	107 (35.5)	50 (35.5)	
Missing	4 (1.3)	0 (0)	
Number of office visits, mean (SD)	5.1 (13.42)	0.7 (1.07)	<0.0001
BMI (kg/m^2^), mean (SD)	26.3 (5.76)	27.6 (5.73)	0.0307
Obesity, *n* (%)	34 (11.3)	37 (26.2)	<0.0001
Illness Duration (yrs), mean (SD)	18.4 (10.98)		
Illness Onset, *n* (%)			
Gradual	104 (34.6)		
Sudden	190 (63.1)		
Missing	7 (2.3)		

SD = Standard deviation; BMI = Body mass index.

**Table 2:** Autonomic symptoms between myalgic encephalomyelitis/chronic fatigue syndrome (ME/CFS) and healthy control (HC) groups.

	ME/CFS (*n* = 301)	HC (*n* = 141)	ES (CI)	*p*-Value
COMPASS-31 score, mean (SD)				
Bladder (0–10)	1.2 (1.62)	0.3 (0.76)	0.67 (0.48 to 0.87)	<0.0001
Vasomotor (0–5)	0.8 (1.31)	0.1 (0.32)	0.68 (0.48 to 0.87)	<0.0001
Secretomotor (0–15)	5.2 (3.61)	1.3 (2.40)	1.20 (0.99 to 1.41)	<0.0001
Gastrointestinal (0–25)	7.6 (4.21)	2.7 (2.95)	1.27 (1.06 to 1.48)	<0.0001
OI (0–40)	16.7 (9.42)	2.5 (5.77)	1.69 (1.47 to 1.91)	<0.0001
Pupillomotor (0–5)	2.4 (1.09)	0.6 (0.83)	1.75 (1.52 to 1.97)	<0.0001
Total (0–100)	34.1 (13.76)	6.8 (8.24)	2.24 (1.98 to 2.50)	<0.0001
Autonomic-related conditions/symptoms ^a^, *n* (%)				
OI abnormalities	101 (33.9)	1 (0.7)	1.07 (0.87 to 1.27)	<0.0001
Other Dysautonomia ^b^	5 (1.7)	0 (0)	0.26 (0.06 to 0.46)	0.1758
Fainting	16 (5.4)	0 (0)	0.47 (0.27 to 0.67)	0.0019
Trouble emptying bladder	22 (7.4)	0 (0)	0.55 (0.35 to 0.75)	0.0001
Malabsorption or chronic diarrhea	23 (7.7)	0 (0)	0.56 (0.36 to 0.76)	0.0002
Low blood pressure	67 (22.5)	5 (3.5)	0.61 (0.41 to 0.81)	<0.0001
Poor appetite	29 (9.7)	0 (0)	0.63 (0.43 to 0.83)	<0.0001
Constipation	84 (28.2)	4 (2.8)	0.78 (0.58 to 0.98)	<0.0001
Dry mouth	82 (27.5)	3 (2.1)	0.81 (0.61 to 1.01)	<0.0001
Irregular heartbeat or palpitations	89 (29.9)	4 (2.8)	0.82 (0.62 to 1.02)	<0.0001
Cold extremities	115 (38.6)	8 (5.7)	0.86 (0.66 to 1.06)	<0.0001
Shortness of breath	55 (18.5)	0 (0)	0.89 (0.69 to 1.09)	<0.0001
Dizziness or vertigo	127 (42.6)	4 (2.8)	1.08 (0.88 to 1.28)	<0.0001
NASA Lean Test ^c^, *n* (%)				
OH	16 (6.2)	4 (3.1)	0.15 (−0.07 to 0.37)	0.1839
POTS	24 (10.6)	6 (4.8)	0.22 (−0.01 to 0.45)	0.0634

SD = Standard deviation; OI = Orthostatic intolerance; POTS = Postural orthostatic tachycardia syndrome; OH = Orthostatic hypotension; ES = Effect size for the differences between groups. Cohen’s d for the differences in COMPASS-31 scores and Cohen’s h for the differences in percentages of having autonomic-related conditions/symptoms and OI abnormalities. Cohen’s effect size is interpreted as a “small” effect (0.2), a “moderate” effect (0.5), a “large” effect (0.8), and a “very large” effect (1.2); CI = 95% confidence interval for the measured ES. ^a^ Abstracted information from medical history. ^b^ Other Dysautonomia include autonomic nervous system disorders, autonomic neuropathy, and dysautonomia, excluding OI. ^c^ OI abnormalities (OH and POTS) were identified using the NASA Lean Test, which was conducted on 390 participants (258 ME/CFS and 131 HC).

**Table 3:** Group means in functioning health measures between ME/CFS participants with and without dysautonomia risk.

	Orthostatic Intolerance Risk (66.8% *)			**Pupillomotor Risk (57.3%)**			**Vasomotor Risk (27.8%)**		
	**Yes (*n* = 189)**	**No (*n* = 94)**	**ES**	**Yes (*n* = 168)**	**No (*n* = 125)**	**ES**	**Yes (*n* = 81)**	**No (*n* = 210)**	**ES**
SF-36 T-Score									
Physical Functioning	30.7 (9.97)	37.3 (11.10)	0.64 ^d^	30.0 (9.79)	36.2 (11.07)	0.60 ^d^	31.0 (10.73)	33.2 (10.7)	0.21
Role Physical	24.7 (8.38)	29.2 (11.33)	0.48 ^c^	24.2 (7.88)	28.6 (11.45)	0.46 ^c^	25.2 (8.76)	26.4 (9.95)	0.13
Bodily Pain	37.1 (10.74)	39.7 (10.49)	0.24	34.5 (9.50)	41.9 (10.92)	0.73 ^d^	35.3 (10.37)	38.7 (10.63)	0.33 ^a^
Vitality	29.7 (7.88)	35.7 (10.86)	0.66 ^d^	29.8 (7.71)	34.0 (10.93)	0.46 ^c^	30.4 (9.10)	32.2 (9.51)	0.20
General Health	28.3 (8.01)	31.9 (10.23)	0.41 ^b^	26.9 (7.44)	32.5 (9.99)	0.65 ^d^	28.8 (8.81)	29.6 (9.04)	0.08
Role Emotional	43.0 (14.51)	45.3 (14.18)	0.16	42.8 (15.15)	45.4 (13.30)	0.17	41.0 (16.02)	45.0 (13.54)	0.28 ^a^
Social Functioning	24.9 (11.09)	31.1 (12.71)	0.53 ^d^	24.7 (10.52)	30.2 (13.27)	0.46 ^c^	26.0 (11.83)	27.8 (11.94)	0.15
Mental Health	44.7 (11.14)	47.4 (10.57)	0.25 ^a^	44.9 (10.90)	46.4 (11.24)	0.14	44.6 (11.28)	46.2 (10.72)	0.14
OGS score	9.5 (4.19)	4.9 (4.07)	1.11 ^d^	9.3 (4.76)	6.8 (4.52)	0.53 ^d^	9.6 (4.80)	7.8 (4.70)	0.39 ^b^
PROMIS T-Score									
Fatigue	68.3 (6.48)	62.3 (8.76)	0.81 ^d^	68.4 (6.06)	63.7 (8.98)	0.63 ^d^	67.5 (8.25)	66.0 (7.42)	0.20
Pain Interference	61.1 (9.01)	58.3 (10.14)	0.31 ^a^	63.0 (8.73)	56.6 (9.39)	0.71 ^d^	62.3 (9.47)	59.3 (9.22)	0.33 ^a^
Pain Behavior	57.6 (7.00)	54.8 (8.78)	0.36 ^b^	58.7 (6.23)	54.3 (8.60)	0.59 ^d^	57.6 (7.80)	56.5 (7.49)	0.14
Sleep Disturbance	59.6 (7.85)	56.1 (7.78)	0.45 ^c^	60.0 (8.16)	56.6 (7.83)	0.42 ^c^	59.2 (8.08)	58.2 (8.08)	0.12
Sleep Related Impairment	62.5 (7.29)	57.1 (7.50)	0.73 ^d^	62.5 (7.70)	58.5 (7.57)	0.51 ^d^	62.0 (8.52)	60.4 (7.59)	0.21
	**Secretomotor Risk (52.7%)**			**Gastrointestinal Risk (66.2%)**			**Bladder Risk (29.8%)**		
	**Yes (*n* = 155)**	**No (*n* = 139)**	**ES**	**Yes (*n* = 188)**	**No (*n* = 96)**	**ES**	**Yes (*n* = 87)**	**No (*n* = 205)**	**ES**
SF-36 T-Score									
Physical Functioning	31.3 (9.83)	33.7 (11.55)	0.23	31.5 (10.08)	34.9 (11.57)	0.31 ^a^	29.9 (10.36)	33.7 (10.88)	0.35 ^b^
Role Physical	25.8 (9.13)	26.2 (10.17)	0.04	25.2 (8.91)	27.7 (10.57)	0.27 ^a^	24.6 (9.07)	26.4 (9.87)	0.19
Bodily Pain	36 (10.12)	39.3 (11.11)	0.31 ^b^	36.4 (10.43)	41.1 (10.50)	0.45 ^c^	34.6 (9.24)	38.8 (11.01)	0.39 ^b^
Vitality	30.6 (9.08)	32.3 (9.20)	0.19	30.7 (8.98)	33.3 (9.77)	0.28 ^a^	30.8 (9.58)	32.1 (9.50)	0.15
General Health	27.9 (8.33)	30.4 (9.16)	0.29 ^a^	27.9 (8.03)	32.1 (9.79)	0.49 ^c^	27.6 (8.66)	30.1 (9.02)	0.28 ^a^
Role Emotional	41.5 (15.44)	46.5 (12.67)	0.35 ^b^	41.4 (14.78)	48.3 (12.36)	0.49 ^c^	42.0 (15.10)	44.3 (14.24)	0.16
Social Functioning	26.7 (11.88)	27.4 (11.90)	0.06	25.4 (11.10)	30.1 (12.81)	0.40 ^b^	25.1 (11.56)	27.8 (11.95)	0.23
Mental Health	44.6 (10.97)	46.6 (10.74)	0.18	43.9 (11.49)	48.6 (9.58)	0.43 ^c^	45.0 (10.01)	45.7 (11.40)	0.06
OGS score	8.4 (4.50)	8.3 (5.13)	0.02	8.8 (4.91)	7.1 (4.40)	0.36 ^b^	9.6 (4.93)	7.7 (4.64)	0.41 ^b^
PROMIS T-Score									
Fatigue	67.2 (7.06)	65.9 (8.27)	0.18	67.5 (7.58)	64.5 (7.39)	0.40 ^b^	67.6 (6.86)	66.0 (8.16)	0.21
Pain Interference	61.5 (8.81)	59.0 (9.98)	0.26 ^a^	61.5 (9.07)	57.4 (9.55)	0.44 ^c^	62.0 (8.32)	59.8 (9.70)	0.24
Pain Behavior	57.8 (7.13)	55.7 (8.05)	0.27 ^a^	57.8 (7.04)	54.8 (8.63)	0.39 ^b^	58.8 (6.00)	56.2 (7.94)	0.35 ^b^
Sleep Disturbance	59.4 (8.10)	57.6 (8.23)	0.22	59.6 (7.73)	56.8 (7.68)	0.36 ^b^	58.4 (7.92)	58.4 (8.21)	0.00
Sleep Related Impairment	61.6 (7.43)	60.2 (8.25)	0.18	62.4 (7.93)	58.0 (6.63)	0.58 ^d^	61.6 (7.83)	60.5 (7.96)	0.14

Note: Values are reported as mean (SD) unless otherwise indicated. * indicates the percentage of being at risk for each domain-specific autonomic dysfunction; ES = Effect size for the mean differences between groups. Cohen’s d for the differences in scores and is interpreted as a “small” effect (0.2), a “moderate” effect (0.5), a “large” effect (0.8), and a “very large” effect (1.2); ^a^ *p* < 0.05, ^b^ *p* < 0.01, ^c^ *p* < 0.001, ^d^ *p* < 0.0001 for the difference in mean scores between ME/CFS participants with and without dysautonomia risk.

**Table 4:** Group means in symptom scores between ME/CFS participants with and without dysautonomia risk.

	**Orthostatic Intolerance Risk**			**Pupillomotor Risk**			**Vasomotor Risk**		
	**Yes (*n* = 189)**	**No (*n* = 94)**	**ES**	**Yes (*n* = 168)**	**No (*n* = 125)**	**ES**	**Yes (*n* = 81)**	**No (*n* = 210)**	**ES**
Sore Throat	2.0 (3.14)	0.8 (1.95)	0.42 ^c^	1.9 (3.22)	1.1 (2.18)	0.30 ^b^	2.3 (3.20)	1.3 (2.68)	0.33 ^a^
Tender Lymph Nodes	3.4 (4.54)	1.9 (3.26)	0.35 ^b^	4.1 (4.83)	1.5 (2.82)	0.64 ^d^	3.5 (4.55)	2.8 (4.22)	0.15
Diarrhea	1.5 (3.02)	0.5 (1.80)	0.38 ^c^	1.6 (3.02)	0.6 (2.00)	0.39 ^c^	1.6 (3.07)	1.0 (2.41)	0.22
Fatigue After Exertion	11.3 (5.34)	7.8 (6.16)	0.63 ^d^	11.6 (5.15)	8.4 (6.25)	0.57 ^d^	11.0 (5.53)	10.0 (5.92)	0.17
Muscle Aches and Pains	7.6 (5.58)	6.3 (4.91)	0.24	8.8 (5.14)	5.2 (5.01)	0.70 ^d^	8.1 (5.71)	6.9 (5.20)	0.23
Joint Pain	5.6 (5.34)	3.4 (4.35)	0.43 ^c^	6.0 (5.39)	3.2 (4.29)	0.56 ^d^	6.2 (5.36)	4.4 (5.00)	0.34 ^a^
Unrefreshing Sleep	10.0 (5.29)	7.5 (5.56)	0.46 ^c^	10.1 (5.33)	8.2 (5.54)	0.34 ^b^	10.1 (5.38)	8.8 (5.44)	0.24
Sleeping Problems	8.4 (5.42)	6.5 (5.27)	0.36 ^b^	9.0 (5.58)	6.3 (4.85)	0.50 ^d^	8.6 (4.98)	7.5 (5.54)	0.21
Headaches	4.5 (4.48)	2.9 (3.73)	0.38 ^b^	4.8 (4.44)	3.2 (4.04)	0.37 ^b^	5.2 (4.78)	3.6 (3.96)	0.38 ^a^
Memory Problems	5.2 (5.15)	3.9 (4.65)	0.26	6.1 (5.01)	3.2 (4.47)	0.59 ^d^	5.3 (5.20)	4.7 (4.99)	0.12
Concentration Problems	6.7 (5.57)	4.8 (4.90)	0.34 ^b^	7.5 (5.40)	4.3 (4.80)	0.64 ^d^	6.7 (5.58)	5.8 (5.28)	0.16
Nausea	2.0 (3.46)	1.0 (2.68)	0.30 ^a^	2.3 (3.89)	1.0 (1.89)	0.41 ^c^	2.4 (3.32)	1.5 (3.25)	0.27 ^a^
Stomach or Abdominal Pain	3.0 (4.50)	1.7 (3.09)	0.32 ^b^	3.1 (4.39)	1.8 (3.46)	0.32 ^b^	3.8 (4.79)	2.1 (3.69)	0.44 ^b^
Sinus or Nasal Problems	4.0 (4.41)	3.4 (4.99)	0.13	4.5 (4.83)	2.8 (4.28)	0.37 ^b^	4.1 (4.77)	3.6 (4.59)	0.11
Shortness of Breath	2.4 (3.93)	1.4 (3.30)	0.28 ^a^	2.9 (4.30)	1.2 (2.84)	0.45 ^c^	2.7 (4.31)	1.8 (3.47)	0.25
Sensitivity to Light	5.4 (5.38)	3.8 (4.97)	0.32 ^a^	7.4 (5.48)	1.7 (2.77)	1.26 ^d^	6.8 (5.74)	4.3 (5.04)	0.49 ^c^
Depression	2.7 (4.54)	1.2 (2.55)	0.37 ^c^	2.2 (3.95)	2.2 (4.16)	0.01	2.7 (4.50)	2.0 (3.86)	0.15
	**Secretomotor Risk**			**Gastrointestinal Risk**			**Bladder Risk**		
	**Yes (*n* = 155)**	**No (*n* = 139)**	**ES**	**Yes (*n* = 188)**	**No (*n* = 96)**	**ES**	**Yes (*n* = 87)**	**No (*n* = 205)**	**ES**
Sore Throat	2.0 (3.20)	1.2 (2.44)	0.26 ^a^	1.9 (2.97)	1.1 (2.65)	0.25 ^a^	2.1 (3.16)	1.3 (2.43)	0.30 ^a^
Tender Lymph Nodes	3.7 (4.73)	2.2 (3.60)	0.35 ^b^	3.0 (4.22)	2.5 (4.20)	0.12	3.9 (4.94)	2.5 (3.79)	0.35 ^a^
Diarrhea	1.3 (2.86)	1.1 (2.46)	0.10	1.6 (3.06)	0.2 (0.71)	0.56 ^d^	1.7 (3.27)	1.0 (2.25)	0.27
Fatigue After Exertion	10.8 (5.66)	10.0 (5.87)	0.14	11.1 (5.47)	8.7 (5.98)	0.44 ^c^	11.0 (5.66)	10.1 (5.84)	0.15
Muscle Aches and Pains	8.2 (5.37)	6.3 (5.17)	0.36 ^b^	8.0 (5.19)	5.3 (5.18)	0.53 ^d^	8.6 (4.77)	6.6 (5.47)	0.38 ^b^
Joint Pain	5.8 (5.38)	3.9 (4.69)	0.39 ^b^	5.6 (5.37)	3.1 (4.09)	0.49 ^d^	6.3 (5.03)	4.1 (4.97)	0.45 ^c^
Unrefreshing Sleep	10.0 (5.27)	8.7 (5.54)	0.23	10.2 (5.09)	7.5 (5.73)	0.50 ^c^	9.6 (5.19)	9.1 (5.58)	0.09
Sleeping Problems	8.6 (5.21)	7.2 (5.58)	0.27 ^a^	8.8 (5.35)	6.1 (5.33)	0.51 ^c^	8.4 (5.30)	7.5 (5.44)	0.17
Headaches	4.3 (4.51)	4.1 (4.19)	0.03	4.6 (4.61)	3.2 (3.73)	0.32 ^b^	5.0 (4.40)	3.9 (4.32)	0.27 ^a^
Memory Problems	5.9 (5.05)	4.0 (4.85)	0.37 ^b^	5.4 (5.11)	3.9 (4.49)	0.31 ^a^	5.5 (5.37)	4.7 (4.89)	0.17
Concentration Problems	6.9 (5.34)	5.5 (5.44)	0.27 ^a^	6.9 (5.56)	4.7 (4.67)	0.41 ^b^	7.3 (5.43)	5.7 (5.39)	0.30 ^a^
Nausea	2.2 (3.63)	1.3 (2.80)	0.29 ^a^	1.9 (3.29)	1.3 (3.25)	0.20	2.3 (3.83)	1.5 (3.02)	0.23
Stomach or Abdominal Pain	2.9 (4.17)	2.2 (3.96)	0.16	3.1 (4.31)	1.3 (3.21)	0.45 ^c^	3.1 (4.44)	2.4 (3.87)	0.17
Sinus or Nasal Problems	4.5 (4.85)	3.1 (4.26)	0.30 ^a^	3.8 (4.54)	3.6 (4.82)	0.06	4.7 (5.02)	3.4 (4.48)	0.27 ^a^
Shortness of Breath	2.8 (4.33)	1.5 (3.10)	0.32 ^b^	2.5 (3.99)	1.3 (2.88)	0.34 ^b^	3.2 (4.51)	1.6 (3.22)	0.43 ^b^
Sensitivity to Light	6.0 (5.63)	3.9 (4.81)	0.41 ^c^	5.3 (5.48)	4.2 (5.00)	0.20	5.8 (5.80)	4.6 (5.05)	0.22
Depression	2.3 (3.99)	2.1 (4.00)	0.06	2.8 (4.34)	1.3 (3.28)	0.36 ^b^	2.1 (3.92)	2.3 (4.10)	0.03

Note: Values are reported as mean (SD) unless otherwise indicated. ES = Effect size for the mean differences between groups. Cohen’s d for the differences in scores and is interpreted as a “small” effect (0.2), a “moderate” effect (0.5), a “large” effect (0.8), and a “very large” effect (1.2); ^a^ *p* < 0.05, ^b^ *p* < 0.01, ^c^ *p* < 0.001, ^d^ *p* < 0.0001 for the difference in mean scores between ME/CFS participants with and without dysautonomia risk.

## Data Availability

Restrictions imposed by the data custodians prevent the datasets from being publicly available or provided by the authors. The program codes used in the current study are available from the corresponding author upon reasonable request. Researchers interested in accessing the datasets used in this study should email the CDC’s ME/CFS Program (cfs@cdc.gov) and discuss the next steps for the data request. The ME/CFS program data review committee will grant access after the review and data use agreement are finalized.

## Supplementary Materials

The following supporting information can be downloaded at https://www.mdpi.com/article/10.3390/jcm14176269/s1: Table S1: Characteristic of participants with ME/CFS (*n* = 301)—overall functioning and symptom status at intake.

## Abbreviations

The following abbreviations are used in this manuscript:

ANS	Autonomic nervous system
ASP	Autonomic Symptom Profile
BMI	Body mass index
BPM	Beats per minute
CDC	Centers for Disease Control and Prevention
CDC-SI	CDC Symptom Inventory
COMPASS-31	Composite Autonomic Symptom Scale 31
DBP	Diastolic blood pressure
ES	Effect size
GI	Gastrointestinal
HC	Healthy controls
NASA	National Aeronautics and Space Administration
NMH	Neurally mediated hypotension
MCAM	Multi-site clinical assessment of myalgic encephalomyelitis/chronic fatigue syndrome (ME/CFS)
ME/CFS	Myalgic encephalomyelitis/chronic fatigue syndrome
MFI-20	20-item Multidimensional Fatigue Inventory
OGS	Orthostatic Grading Scale
OH	Orthostatic hypotension
OI	Orthostatic intolerance
PEM	Post-exertional malaise
POTS	Postural orthostatic tachycardia syndrome
PROMIS	Patient-Reported Outcomes Measurement Information System
SD	Standard deviation
SEM	Standard error of mean
SF-36v2	Short Form Health Survey—36-item Version 2
SBP	Systolic blood pressure
