# Supplementary material for: Autonomic Dysfunction in Myalgic Encephalomyelitis/Chronic Fatigue Syndrome (ME/CFS): Findings from the Multi-Site Clinical Assessment of ME/CFS (MCAM) Study in the USA
Source: J Clin Med. 2025 Sep 5;14(17):6269. doi: 10.3390/jcm14176269 (PMC12428951; doi:10.3390/jcm14176269)
Supplement: Supplementary file 1 [file jcm-14-06269-s001.zip › jcm-3806350-supplementary.pdf]

Table S1. Characteristic of Participants with ME/CFS (n=301) – Overall Functioning and Symptom Status at Intake

	Mean	SD	SEM	Minimum	Median	Maximum
**SF-36 Scores (0-100)[a]**						
Physical Functioning	40.05	23.07	1.34	0	40	100
Role Physical	5.62	19.99	1.16	0	0	100
Bodily Pain	40.40	22.92	1.32	0	41	100
Vitality	18.00	16.80	0.98	0	15	100
General Health	26.91	17.45	1.02	0	22	90
Role Emotional	75.45	39.84	2.32	0	100	100
Social Functioning	27.15	23.59	1.37	0	25	100
Mental Health	67.81	19.80	1.15	0	72	100
**PROMIS T-Scores**						
Fatigue	67.43	7.00	0.40	29.4	67.8	83.2
Pain Interference	60.68	9.23	0.53	41.0	61.8	78.3
Pain Behavior	56.72	7.59	0.44	36.7	59.2	68.3
Sleep Disturbance	59.01	7.81	0.45	35.9	58.3	76.5
Sleep Related Impairment	61.40	8.10	0.47	30.0	61.3	80.0
**MFI-20 (4-20)**						
General Fatigue	18.09	2.45	0.14	4	19	20
Physical Fatigue	17.28	2.89	0.17	4	18	20
Reduced Activity	15.84	3.63	0.21	4	16	20
Reduced Motivation	11.83	3.99	0.23	4	12	20
Mental Fatigue	14.40	3.82	0.22	4	15	20
**CDC-SI**						
no. of CFS symptoms (0-8)[b]	5.85	1.93	0.12	0	6	8
CFS symptom score (0-128)[c]	53.60	24.22	1.46	0.0	54.5	115.5

[a] SF-36 scores are listed for the 0-100 rescaled scores to compare with Table 3 by Unger 2017 rather than T-scores presented in Table 3 in this paper.

[b] The score for the number of CFS symptoms ranges from 0–8, with higher scores indicating more symptoms lasting for 6 months or longer.

[c] The 8 CFS symptom score ranges from 0–128, with higher scores indicating higher severity of symptoms.
